# Subperiosteal Ridge Augmentation Technique Utilizing Sticky Bone: A Case Series

**DOI:** 10.7759/cureus.65641

**Published:** 2024-07-29

**Authors:** Kandhal Yazhini P, Nidhita Suresh, G Kaarthikeyan

**Affiliations:** 1 Periodontology, Saveetha Dental College and Hospitals, Saveetha Institute of Medical and Technical Sciences, Saveetha University, Chennai, IND

**Keywords:** ridge defects, quality of life, ridge augmentation, sticky bone, iprf

## Abstract

This case series presents the application of a novel minimally invasive technique for augmenting ridge defects across four edentulous sites, thereby circumventing common postoperative complications associated with conventional ridge augmentation techniques. The case series details three cases encompassing four edentulous sites with class I and III ridge defects. This approach uses a minimally invasive subperiosteal technique for ridge augmentation, followed by a delayed implant placement. Subperiosteal incisions were made mesial to the edentulous sites, and subperiosteal pouches were created using tunneling instruments. Sticky bone (comprising injectable platelet-rich fibrin (IPRF) and xenograft) was applied to the pouches, followed by Vicryl suturing. The cone-beam computed tomography (CBCT) assessed dimensional changes between baseline and 180 days post-ridge augmentation. Subsequently, during implant insertion after 180 days, bone samples were collected, decalcified using 10% formic acid, and sectioned to a thickness of 5 µm. Histological analysis of the bone samples was conducted using a bright field microscope, while histomorphometric analysis was carried out using Image J software. The modified subperiosteal tunneling technique employed in this case report, coupled with using sticky bone as an augmentation material, demonstrates promise as a reliable method for ridge augmentation.

## Introduction

Alveolar ridge resorption defects frequently occur as an inevitable consequence after tooth extraction, posing a significant challenge for implant placement in the edentulous area [1]. Subperiosteal ridge augmentation is a crucial surgical technique designed to enhance the contour and volume of the alveolar ridge in patients with bone deficiencies. This technique involves the elevation of the periosteum and the placement of grafting materials directly on the bone surface beneath the periosteal layer [2]. Sticky bone, a mixture of platelet-rich fibrin (PRF) and bone graft materials, has been utilized to enhance the efficacy of this technique. Its cohesive and moldable properties improve graft stability and promote osteogenesis [3].

Traditional techniques for treating ridge defects often involve flap elevation, which can lead to bone resorption due to compromised vascularization from the supraperiosteal vessels. A crucial factor for successful bone regeneration is maintaining adequate space to facilitate bone ingrowth, typically achieved with a membrane. This case report introduces a novel approach that involves elevating the periosteum and using sticky bone as the augmentation material, thereby eliminating the need for a membrane [4]. Sticky bone, easily shaped and placed into the prepared pouch using a bone graft carrier, simplifies the augmentation process. By omitting the membrane, this method helps prevent common postoperative complications such as flap dehiscence and membrane exposure, often resulting in inadequate bone regeneration.

Additionally, this technique preserves the vascularity of the flap from the supraperiosteal vessels by elevating the periosteum without completely stripping it from the bone. Maintaining this blood supply is essential for the healing process and significantly enhances the overall success of the augmentation [5]. By preserving the vascular integrity of the flap, the technique ensures better nourishment and support for the regenerating bone, promoting more effective and reliable outcomes. Following the augmentation, bone samples were obtained using a trephine during implant insertion. These samples underwent histological and histomorphometric analyses using ImageJ software (National Institutes of Health, Bethesda, Maryland) to assess the quality and quantity of new bone formation. The findings demonstrated the effectiveness of this technique in promoting bone regeneration, providing a promising alternative for horizontal ridge augmentation without the complications associated with membrane use [6,7].

## Case presentation

This article presents findings from three cases (involving four edentulous sites) with class I and class III ridge defects. A minimally invasive subperiosteal technique was employed for ridge augmentation before implant placement. The patients included two individuals with class I Seibert's ridge defects (in two edentulous sites) and one with class III Seibert's ridge defects (also in two edentulous sites). All patients were systemically healthy, aged between 40 and 58, with a gingival thickness exceeding 2 mm. Ethical clearance was obtained from the Scientific Review Board (SRB/SDC/PERIO-1989/23/076).

Subperiosteal minimally invasive ridge augmentation (SMIRA) is a surgical technique used for ridge augmentation. This procedure employs magnifying loupes with 2× magnification for precision. It begins with the administration of local anesthesia, followed by a subperiosteal vertical incision made with an SM63 microsurgical blade adjacent to the augmentation site. Subperiosteal tunneling is performed using TKNIX and TKN2X tunneling knives to create a subperiosteal pouch [3]. The decortication process involves a back-action chisel. Sticky bone is then placed into the subperiosteal pouch, which is made by mixing 10 mL of venous blood-derived liquid i-PRF (700 rpm, 60 g for three minutes) with xenogenic particulate bone (Bio-Oss, granule size 1-2 mm) (Geistlich Pharma AG, Wolhusen, Switzerland) [4,5]. The area is sutured using 5-0 Vicryl sutures (Ethicon, Bridgewater, New Jersey). Six months after augmentation, bone samples were collected using a trephine during implant placement and preserved for histological analysis. Cone-beam computed tomography (CBCT) scans are employed to evaluate changes in ridge width and height.

Case 1

**Figure 1:**
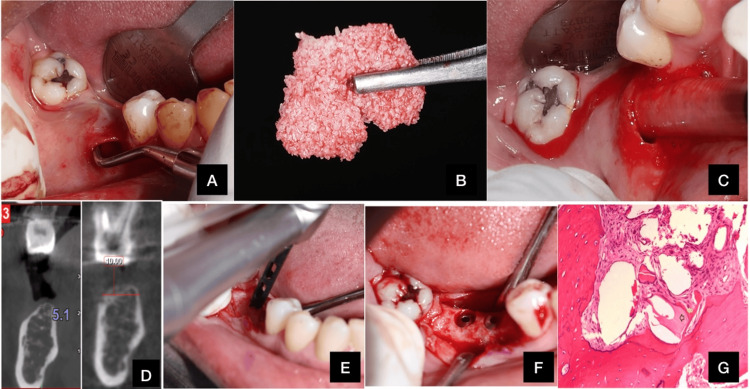
(A) Subperiosteal pouch prepared; (B) sticky bone; (C) sticky bone placed into the subperiosteal pouch; (D) CBCT measurements; (E) bone sample obtained; (F) implant placed; (G) histologic section under 100× magnification

A 56-year-old systemically healthy female with a class I Seibert's ridge defect and gingival thickness exceeding 2 mm underwent the ridge augmentation procedure. Using the aforementioned technique, the surgery was successfully performed, and histological samples were collected for analysis, as shown in Figure 1.

Case 2

**Figure 2:**
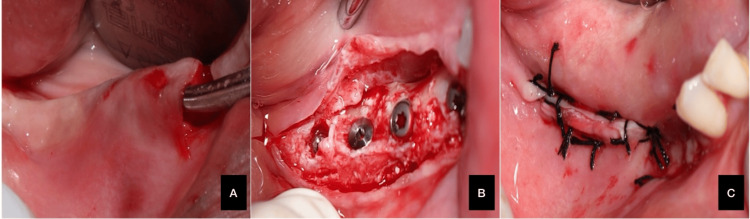
(A) Subperiosteal pouch prepared; (B) implant placed; (C) sutures placed

**Figure 3:**
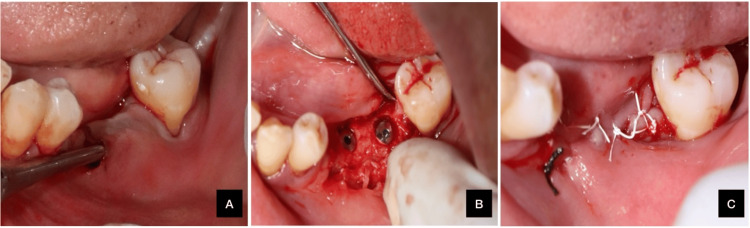
(A) Subperiosteal pouch prepared; (B) implant placed; (C) sutures placed

A 58-year-old systemically healthy female with class III Seibert's ridge defects on both sides and gingival thickness exceeding 2 mm underwent SMIRA using the same technique as in Case 1. The procedure was completed successfully, and histological samples were collected, as shown in Figures 2, 3.

Case 3

**Figure 4:**
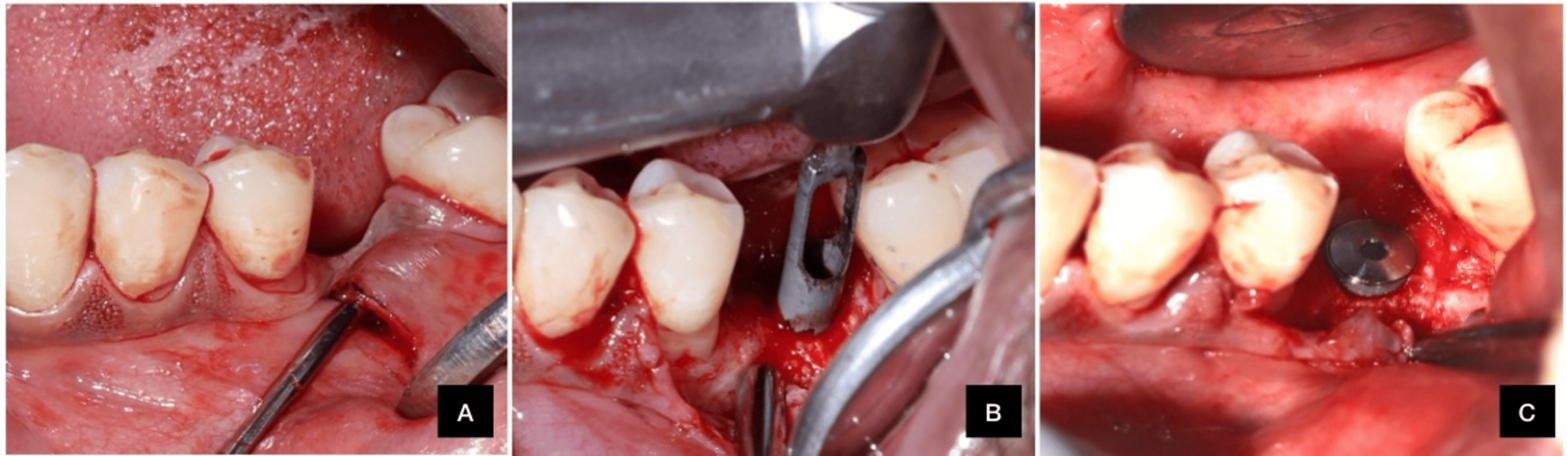
(A) Subperiosteal pouch prepared; (B) bone sample obtained; (C) implant placed

A 58-year-old systemically healthy male with a class I Seibert's ridge defect and gingival thickness exceeding 2 mm also underwent SMIRA using the same protocol as in Case 2. The procedure was successful, and histological samples were collected, as illustrated in Figure 4.

CBCT scans were used to assess dimensional changes in ridge width and height between baseline and 180 days post-augmentation, maintaining consistent settings of 400 μm voxel size, 90 kV, and 10 mA, with a scan time of 13.9 seconds. The analysis revealed significant increases in width and height, as shown in Table 1.

**Table 1:** Ridge width at baseline and after six months

Seibert's Classification	Ridge Width (Baseline)	Ridge Width (After Six Months)	Ridge Height (Baseline)	Ridge Height (After Six Months)
Case 1: site 1 (class 1 ridge defect)	3.3 mm	8.4 mm	nil	nil
Case 1: site 2 (class 3)	5.1 mm	10 mm	9.0 mm	11.6 mm
Case 2: site 3 (class 3)	3.0 mm	5.2 mm	9.6 mm	12.11 mm
Case 3: site 4 (class 1)	3.8	6.1	nil	nil

Bone samples underwent decalcification using 10% formic acid and were sectioned to a thickness of 5 µm. Slides were stored using hematoxylin and eosin and analyzed histologically. Histological examination indicated the presence of remnant particles resembling osseous tissue fragments, with pale eosinophilic staining and empty cavities. Newly formed bone regions exhibited osteocytes within cavities and more pronounced eosinophilic staining. The remaining tissue comprised connective tissue with fibroblasts, collagen fibers, and tiny capillaries [6].

Histomorphometric analysis using ImageJ on eight selected sections revealed 69.45% (±0.09%) newly formed bone, 27.89% (±0.07%) connective tissue, and 2% (±0.02%) remnant graft material. These findings were obtained by overlaying a grid of 2000 pixels onto histological sections magnified at 10× and performing a point counting procedure to determine the percentage of bone, connective tissue, and remaining graft particles relative to the total area, as shown in Table 2 [7].

**Table 2:** Percentage of bone, connective tissue, and remnant graft particles by histomorphometric analysis

Results of the Histomorphometric Analysis Taken From the Center of the Bone Trephine Sample			
Seibert’s classification	Percentage of bone	Percentage of connective tissue	Percentage of remnant graft particles
Case 1: site 1 (class 1 ridge defect)	72.5%	27.5%	nil
Case 2: site 2 (class 3)	58.5%	41.4%	0%
Case 2: site 3 (Class 3)	79.6%	20.3%	nil
Case 3: site 4 (Class 1)	83.08%	16.9%	0.02%
Results of the histomorphometric analysis taken from 0.5 mm coronal to the center of the bone trephine sample			
Seibert’s classification	Percentage of bone	Percentage of connective tissue	Percentage of remnant graft particles
Case 1: site 1 (class 1 ridge defect)	68.7%	31.2%	nil
Case 2: site 2 (class 3)	78.04%	21.9%	nil
Case 2: site 3 (class 3)	61.5%	32.6%	5.9%

## Discussion

The conventional techniques for bone augmentation often face significant challenges, including the need for flap elevation, placement of cell occlusive membranes, and the risk of wound dehiscence. These factors can complicate the procedure and increase the likelihood of postoperative complications. To address these issues, this case report investigates the use of the subperiosteal tunneling technique for horizontal augmentation of posterior edentulous ridges [8,9]. This approach offers promising results, reducing postoperative discomfort and complications, which are common with traditional methods. The subperiosteal tunneling technique involves creating a subperiosteal pouch without the need for extensive flap elevation. This minimizes tissue trauma and preserves the periosteum's integrity, which is crucial for bone regeneration. The technique has shown encouraging outcomes in ridge augmentation, making it a viable alternative to conventional methods [10].

In this case series, sticky bone was chosen as the augmentation biomaterial. Sticky bone combines the osteoconductive properties of demineralized bovine bone mineral (DBBM) with the benefits of growth factor-mediated activation and differentiation of progenitor cells. This composite material promotes bone growth and provides a stable scaffold for new bone formation [11]. Additionally, DBBM's slower substitution rate is advantageous for maintaining the augmentation volume during the consolidation phase. This slow resorption allows for a more gradual replacement with native bone, ensuring better integration and long-term stability of the augmentation site [12,13]. By using sticky bone, the procedure harnesses the dual benefits of immediate structural support and enhanced biological activity, making it an effective choice for horizontal ridge augmentation in posterior edentulous areas [14].

In this case series, barrier membranes were intentionally not used in conjunction with sticky bone for managing horizontal ridge defects. Instead, sticky bone was solely relied upon to contain the graft material within the subperiosteal pouch, effectively addressing the defect area. To reduce the risk of excessive tension on the mucosa, which could lead to dehiscence and exposure of graft sites, patients with a minimum gingival thickness of 2 mm were specifically included in the study [15]. Previous studies have demonstrated the efficacy of similar techniques. In 2017, Lee showed a ridge width gain of 6.47 mm using the Subperiosteal Minimally Invasive Aesthetic Ridge Augmentation Technique (SMART) [16]. Similarly, in 2018, Kakar et al. reported horizontal ridge width gains ranging from 4.17 mm to 8.56 mm in nine patients who underwent lateral ridge augmentation using the tunneling technique and Easy-Graft Crystal (a composite of β-tricalcium phosphate and hydroxyapatite) [17].

Traditionally, ridge augmentation has relied on xenografts combined with barrier membranes to address horizontal and vertical ridge defects. The membrane was believed to prevent progenitor cell migration and angiogenesis by acting as a physical barrier to chemotaxis [12]. However, recent findings suggest that a different approach may be beneficial. Progenitor cells in the periosteum play a crucial role in bone defect repair due to their osteogenic potential, and the absence of a barrier membrane allows for enhanced migration and activity of these cells. These insights underscore the potential of the subperiosteal tunneling technique combined with sticky bone in promoting effective ridge augmentation. This method not only simplifies the procedure by eliminating the need for barrier membranes but also leverages the natural regenerative capacity of periosteal progenitor cells, offering a promising alternative for managing horizontal ridge defects [18].

While growth factors typically negate the need for decortication in grafting procedures, back-action chisels were employed for decortication in this case series. This approach promotes bleeding, facilitates progenitor cell migration to the graft site, and enhances mechanical interlocking between the graft and recipient site [19]. In this case series, the composition comprised 69.45% (±0.09%) bone, 27.89% (±0.07%) connective tissue, and 2% (±0.02%) remnant graft particles. This contrasts with the findings of Lee, who reported 50% bone, 47% bone marrow or fibrous tissue, and no residual bone graft material when using rhPDGF-BB + particulate bone matrices [16]. The accelerated turnover rate of the bone graft and the minimal percentage of residual bone graft observed in this study may be credited to the growth factor concentrates found in the sticky bone. These concentrates stimulate osteoprogenitor cells, promoting faster bone formation [20]. Kakar et al. noted 32.9% bone, 22.4% connective tissue, and 44.7% easy graft (β TCP+ HA) [17]. Differences between their results and those of the present case series may stem from variations in the biomaterials utilized for augmentation and the duration of follow-up. In our study, we observed a minimal presence of xenogenic or residual graft particles. However, it is important to acknowledge the limitations of this study, which include a small number of subjects and a narrow age range of participants. Additionally, this case series demonstrated that the technique was more effective in treating horizontal defects but less effective for vertical ridge defects.

## Conclusions

The findings of this case report strongly suggest that the modified subperiosteal tunneling technique, when combined with sticky bone, is a reliable and effective method for ridge augmentation. Sticky bone, used as the augmentation material, demonstrated significant benefits in promoting new bone formation. Its osteoconductive properties, along with its ability to support progenitor cell activity and growth factor release, contribute to a stable and conducive environment for bone regeneration. This technique not only simplifies the augmentation process by eliminating the need for barrier membranes but also leverages the natural regenerative capabilities of the periosteum, making it a promising approach for horizontal ridge augmentation.
